# Substance use patterns among sexual minority women: a mini review

**DOI:** 10.3389/fgwh.2026.1758747

**Published:** 2026-05-04

**Authors:** Swapnajeet Sahoo, Arshia Sood

**Affiliations:** Department of Psychiatry, Post Graduate Institute of Medical Education and Research (PGIMER), Chandigarh, India

**Keywords:** LGBTQ, Lesbian, pattern, sexual minorities, substance & alcohol use, women

## Abstract

**Background:**

Sexual minority women (SMW), including lesbian, bisexual, and other non-heterosexual women, experience higher rates of substance use (SU) and substance use disorders (SUDs) than heterosexual women, yet remain relatively underrepresented in the literature.

**Objectives:**

This structured mini-review aimed to synthesize evidence on prevalence patterns, determinants, intersectional influences, treatment barriers, and intervention approaches related to substance use among SMW.

**Methods:**

A structured narrative review of epidemiological studies, reviews, and meta-analyses was undertaken using predefined thematic domains: epidemiology, determinants/pathways, intersectionality, and treatment-related issues. Evidence was synthesized narratively because of heterogeneity in populations, outcome measures, and study designs.

**Results:**

Across much of the available literature, SMW show elevated prevalence of alcohol, tobacco, cannabis, and polysubstance use compared with heterosexual women, with bisexual women frequently identified as the highest-risk subgroup. Reported determinants include minority stress-related processes, internalized stigma, discrimination, victimization, and adverse childhood experiences. Intersectional disadvantage, including racial/ethnic minority status and socioeconomic marginalization, may further amplify vulnerability. Treatment access is hindered by stigma, limited LGBTQ+-affirming services, and gaps in provider cultural competence. Evidence for SMW-specific interventions remains limited.

**Conclusions:**

Available evidence suggests that substance use disparities among SMW are shaped by minority stress, intersecting social disadvantage, and barriers to inclusive care. More methodologically rigorous, intersectionally informed, and geographically diverse research is needed, alongside development of identity-affirming and trauma-informed interventions.

## 1 Introduction

Sexual identity and gender identity are distinct concepts that are frequently confused. Sexual identity is a person's identity based on their romantic or physical attraction. Conversely, gender identity is a person's physiological sense of being a specific gender, such as man or woman, neither gender, or a combination of these. Gender identity is classified as cisgender (someone who acknowledges their physiological identity as being identical with their assigned gender at birth) or transgender (someone who recognizes their physiological identity as being different from their assigned gender at birth). Nowadays, sexual and gender minority identities are frequently recognized in tandem, these being, lesbian, gay, bisexual, transgender, queer, questioning, and other emerging sexual (and gender) identities, which are commonly referred to as LGBTQ +  (1).

Compared to heterosexual populations, sexual minorities have a higher risk of substance use (SU) and substance use disorders (SUD). Sexual minority women (SMW) appear to be disproportionately affected in much of the available literature with these higher rates of SU and SUDs, whereas results for sexual minority men are more inconsistent (2–5). Despite being a part of the broader LGBTQ + research, SMW are often underrepresented, which obscures female-specific drug use patterns and creates gaps in knowledge about their unique risks.

## 2 Objectives

The present review was guided by four review questions concerning prevalence, determinants, intersectional variation, and treatment-related issues among SMW. This mini-review aimed to synthesize evidence on: (1) prevalence and patterns of substance use and substance use disorders among sexual minority women;(2) determinants and explanatory pathways, particularly minority stress-related processes;(3) intersectional and sociodemographic variations in risk; and (4) treatment barriers and available intervention approaches.

## 3 Methodology

This mini-review used a structured narrative review approach to synthesize evidence on substance use and substance use disorders among sexual minority women (SMW). The review aimed to address the following questions:

1. What patterns of alcohol, tobacco, cannabis, polysubstance use, and substance use disorders have been reported among SMW?
2. What psychosocial and structural determinants have been associated with these outcomes?
3. How do intersectional factors such as race/ethnicity and socioeconomic position shape risk?
4. What treatment barriers and intervention approaches have been described for SMW?

A literature search was conducted in PubMed, Scopus, PsycINFO, and Google Scholar for studies published in English. Search terms included combinations of: “sexual minority women,” “lesbian,” “bisexual women,” “women who have sex with women,” “substance use,” “substance use disorder,” “alcohol,” “tobacco,” “cannabis,” “polysubstance use,” “minority stress,” “intersectionality,” “treatment,” and “barriers.” To ensure comprehensive coverage, reference lists of key review articles and relevant retrieved papers were also hand-searched for additional sources. We prioritized epidemiological studies, systematic/scoping reviews, and meta-analyses relevant to prevalence, determinants, treatment access, and interventions.

Titles and abstracts of retrieved records were screened for relevance to the review objectives, followed by full-text assessment of potentially eligible articles. Studies were considered for inclusion if they: (a) reported findings specifically relating to SMW or presented subgroup analyses permitting interpretation of SMW-related outcomes;(b) addressed substance use patterns, substance-related harms, determinants, treatment barriers, or intervention strategies; and (c) were empirical studies, systematic/scoping reviews, or meta-analyses. Studies focusing exclusively on sexual minority men, opinion pieces without substantive data synthesis, and articles lacking direct relevance to the review objectives were excluded. Article eligibility and thematic relevance were discussed among the authors during manuscript preparation to ensure consistency in study selection and interpretation.

As this review was conducted as a structured narrative mini-review rather than a formal systematic review, quantitative study selection metrics (e.g., exact numbers of records screened and excluded at each stage) were not prospectively recorded.However, study identification and selection were guided by predefined thematic relevance and eligibility criteria to ensure methodological transparency and conceptual comprehensiveness.

Evidence was narratively synthesized under four predefined thematic domains—epidemiology, determinants/pathways, intersectionality/sociodemographic variation, and treatment access/interventions—which were selected *a priori* based on the primary objectives of the review and recurring conceptual domains identified during preliminary literature appraisal.Owing to heterogeneity in study populations, definitions of sexual orientation, outcome measures, and study methodologies, quantitative pooling or meta-analysis was not undertaken.

### 3.1 Consideration of evidence quality

Given the mini-review design and inclusion of heterogeneous evidence sources, a formal risk-of-bias scoring tool was not applied. However, the interpretation of findings considered key indicators of evidence strength, including study design, sample size, representativeness, consistency of findings across studies, and whether results were derived from population-based data, longitudinal designs, or review-level syntheses. Particular caution was applied to findings from cross-sectional studies, convenience samples, and studies conducted primarily in U.S. settings, as these may limit causal inference and generalizability.

### 3.2 Theoretical framework

To understand why these risks are especially associated with sexual minorities, two explanatory theoretical constructs have been proposed – Minority Stress Theory (6) and Intersectionality Theory. Together, these frameworks help explain both the external pressures and internalized challenges that influence patterns of substance use among SMW.

### 3.3 Minority stress theory

According to the minority stress theory, first given by Meyer in 2003, health inequalities between heterosexual and sexual minority groups are caused by the extra social stress that these groups endure due to their stigmatized social status. This stress has been referred to as “minority stress” and is distinguished from “general stress” that any of us may face in our daily lives. This theory further describes both distal and proximal stress processes (7).

Distal minority stressors include those that come from individuals or organizations that affect LGBTQ + people, such as unfair laws and regulations and events coloured with stigma and discrimination. These could be acute—including events which could be minor (like being treated disrespectfully) or major (like losing one's job or experiencing violence). Chronic stressors, such as poverty, may also be among them.

Proximal minority stressors include internalized stigma, which is the belief that one is “less than others” simply by virtue of one's identity; perceived stigma, which is the expectation that one will be treated differently because one is aware of the prevailing social stigma. This may lead to an attempt to conceal one's LGBTQ + identity to defend oneself against the distal minority stressors.

Against these detrimental stresses, there exist the coping mechanisms (both individual and collective), resilience and social efforts to thwart such practices in the community. Hence, the overall influence on the health of the sexual minorities is determined by the net effect of both opposing forces at play.

Building upon this framework, the next theory further expands our understanding by examining how multiple social identities interact to influence risk and resilience in SMW.

### 3.4 Intersectionality theory

The theoretical framework of intersectionality, introduced by Kimberlé Crenshaw in 1990s, is a paradigm for understanding how a person's experiences of privilege and oppression are shaped by the interaction and overlap of several facets of their social identity, including gender, sexual orientation, race, socio-economic status, disability, and religion. It emphasizes that people who are marginalized by several oppressed groups frequently experience distinct, compounded kinds of disadvantage that are missed by single-axis studies (e.g., focusing only on race or gender or sexual orientation/identity) (8).

Throughout history, women, especially those from marginalized backgrounds (including SMW), have been disproportionately impacted by intersecting inequalities, such as poverty, violence, and discrimination at the workplace. Intersectionality has been used in a lot of empirical research and policy work to address historical exclusions of women's experiences. In domains such as public health, psychiatry, and substance use, concentrating on women reveals how gender norms interact with stigma, sexuality, and social roles to influence outcomes differently.

A complementary syndemic perspective further posits that these intersecting disadvantages may cluster and interact synergistically with co-occurring psychosocial burdens such as trauma, depression, victimization, and structural marginalization, thereby magnifying vulnerability beyond the cumulative sum of individual risk factors.

### 3.5 Epidemiology of SUDs in sexual minorities

Numerous population-based studies have generally shown that SMW exhibit higher rates of substance use and related disorders than heterosexual women. The table below summarizes key studies exploring prevalence patterns across different samples, age groups, and types of substances used (Table 1).

**Table 1:** Epidemiological studies on SUDs in sexual minorities.

Study, Year, Country	Population	Study Sample	Age	SU/SUDs examined	Findings
Hahm et al. (2008) (9), USA	AAPIs	1,108	Adolescence- young adulthood	Tobacco, binge drinking, cannabis, other SU	Increased use in young adulthood, highest prevalence and risk of SU in AAPI SMW than heterosexual women & men, and SMM
McCabe et al. (2009) (4), USA	Heterosexuals, 2% LGB; 4% with same-sex partners, 6% with same-sex attraction	34,653	>20 years	Alcohol, marijuana and other SU	Non-heterosexual orientation was linked to higher risk of SU & dependence. Risk patterns were more pronounced among women
Hughes et al. (2009) (10)				Alcohol and other drug use/ dependence	Strong associations between victimization of SMM&W (especially childhood neglect) and any past-year SUDs
Marshal et al. (2012) (11), USA	Heterosexuals & 6% with same-sex orientation/ identity/ attraction	527 females	17 years	Tobacco and alcohol use	SMW reported higher past-year SU rates
Goldberg et al. (2013) (12), USA	Heterosexuals and SMGs	14,152	24–32 years	Tobacco, alcohol, marijuana & other substance abuse/ dependence	SMW are more likely than heterosexual women to experience all SU/SUDs
Estrich et al. (2014) (13), USA	75.4% heterosexuals, 9.3% lesbians, 15.3% bisexuals	669	16–66 years	Alcohol & SU	SMW had twice the odds of SU in the last year, bisexual women had the highest odds of SU overall
Dermody et al. (2016) (14), USA	Heterosexuals and SMGs (8% - lesbians/ bisexuals)	2,064 females	17 years	Alcohol, cigarette, & marijuana use	SMWs had an increased risk for SU than heterosexuals
Kerridge et al. (2017) (3), USA	Heterosexuals and SMGs (1.5% gays/lesbians, 1.3% bisexuals)	36,309	≥18 years	Alcohol, tobacco & other SUDs	SUDs higher in SMGs, bisexual followed by lesbian women showed the highest risk across nearly all SUDs
Caputi (2018) (15), USA	Heterosexuals and SMGs	15,624	Adolescents	Alcohol, tobacco, marijuana, prescription drug & illicit drug use	SMW, especially bisexuals, had increased risk
Dermody (2018) (16), USA	Heterosexuals and SMGs	15,624	14–18 years	Alcohol, tobacco, marijuana use, & binge drinking	SMW, especially bisexuals, had increased risk for all SUDs & polysubstance use
Schuler et al. (2018) (17), USA	Heterosexuals and SMGs	67,354	18–49 years	Alcohol, tobacco, marijuana, illicit drug, SUD & binge drinking	Bisexual women: higher risk at all ages; lesbians/gays: higher risk in youth only
Boyd et al. (2019) (18), USA	Heterosexuals and SMGs	36,309	≥18 years	Alcohol, tobacco & SUD	Higher prevalence & severity in bisexual & “not sure” women
Talley et al. (2019) (19), USA	Heterosexuals and SMGs	3,48,175	14–18 years	Alcohol, tobacco, marijuana use	Bisexual girls > lesbians at higher risk for early onset & persistent use of tobacco & marijuana
Dermody et al. (2019) (20), USA	Heterosexual and SMGs (17% lesbian/ bisexual)	2,263 females	13–20 years	Alcohol, cigarette, & marijuana use	SMW had higher frequencies of all SU across the entire age continuum, especially early adolescence
Schuler & Collins (2020) (5), USA	Heterosexuals and LGBs	1,26,463	≥17 years	Alcohol, tobacco, marijuana, illicit drug, opioid misuse, SUD & binge drinking	SMM & SMW had higher risks than heterosexuals. Bisexual women had higher risks than lesbians
Ehlke et al. (2022) (21), USA	Heterosexuals and 18.8% SMW	3,020 females	≥18 years	Alcohol, tobacco, marijuana use	SMW had higher prevalence of cannabis and polysubstance use than heterosexuals

USA, United States of America; AAPI, Asian American and Pacific Islander; SU, Substance use; SMW, Sexual Minority Women; SMM, Sexual Minority Men; LGB, Lesbian, Gay, Bisexual; SUDs, Substance Use Disorders, SMGs, Sexual Minority Groups.

Overall, the epidemiological evidence demonstrates that SMW, especially bisexual women, are at disproportionately higher risk of alcohol, tobacco, and drug use across adolescence and adulthood. These recurring findings underline the need to explore why such disparities persist.

### 3.6 Determinants and pathways of substance use

There are various determinants of SU and SUDs in SMW. Minority stress, i.e., proximal and distal LGBTQ + stressors and the use of substances as a coping mechanism to counteract the social isolation are widely recognised as being a driving force behind the SU/SUD patterns in sexual minorities, including SMW (22–29). Internalized heterosexism, i.e., the internalization of negative societal attitudes on non-heterosexual identity and orientation, is one stressor that has drawn a lot of attention and is partially, if not fully, associated with AUD/SUD (30, 31).

Additionally, being “out” to more people correlates with higher SU, possibly due to greater exposure to prejudice or social rejection (22), especially for bisexual women (32). In other studies, non-disclosure of sexual orientation is linked with higher illicit drug use (33). Other recognized factors are sociocultural variables such as the widespread availability of drugs within the LGBTQ + communities (23) and discrimination (23, 25, 28, 33–35).

In a scoping review, adverse childhood experiences was considered a risk factor for SU/SUDs (36). A systematic literature review outlined childhood sexual abuse as being a potential risk factor for adverse mental health outcomes and SUDs in SMW (37).

In a meta-analysis by Goldbach et al. (2014) (38), various minority stressors were noted to be linked to SU, the strongest of these being victimization (24, 25, 27, 33, 39), lack of a supportive environment, externalizing behaviours like conduct problems and truancy, internalizing behaviours such as depression and anxiety, psychological stress (general and gay-related stress), facing rejection after coming out, and homelessness.

In general population, SU is known to decrease as age advances, however, this is not always true for sexual minorities (24). Even though younger LGBTQ + adults display heavier alcohol and drug use, evidence suggests that older age is less pronounced as protective a factor as it is in heterosexuals (22, 28, 33). Some evidence also suggests that alcohol use may elevate as age advances in lesbian/bisexual women (40). This may be due persistent stressors, social isolation or delayed social milestones like marriage/parenthood.

In heterosexual communities, men tend to have greater incidence of SU/SUD; however, this gender disparity is less noticeable in sexual minority groups. Lesbian/bisexual women have comparable or higher rates of alcohol use than gay/bisexual men (3, 41). This is termed as the gender paradox (24). On the contrary, gay/bisexual men are more likely to engage in club drug/illicit drug use (22). This has been hypothesized to be due to weaker adherence to traditional gender norms within sexual minority communities.

Bisexuality has consistently emerged as a standalone risk factor for highest levels of alcohol and drug use across studies (4, 15, 18, 22). This persists even after controlling for openness about sexual orientation. Possible explanations for this include minority stress from both sides, i.e., heterosexual and gay/lesbian communities (3, 18, 42), having lower social support (43), and a mismatch between sexual identity and behaviour (18, 22).

SU/SUDs, including club drug use, have also been linked to high involvement in gay culture, i.e., frequent attendance at bars, sex clubs, and bathhouses, especially among men who have sex with men (MSM). This may be attributed to peer influence which reinforces SU (22–24, 27, 28, 33). Higher community involvement was notably a risk factor for SU in bisexual women, but not for lesbians or queer women (32, 44). On the other hand, some studies have notes that community belonging can act as a protective factor by providing social support (27, 28).

There appears to be a bidirectional link between HIV/AIDS (human immunodeficiency virus/acquired immunodeficiency syndrome) and SU/SUD. SU increase high risk behaviours and being HIV-positive predicts higher poly-substance use and club drug use. SU may serve as a coping mechanism for HIV-related stress, stigma, or emotional distress (22).

It has been seen that countries with anti-LGBT laws show higher AUD rates among sexual minorities. Legal protection and acceptance correlate with better mental health outcomes (24, 27, 33).

Religious affiliation has been shown to be protective for heterosexuals but not for SMW (33, 45).

### 3.7 Intersectionality and sociodemographic variations

According to the Intersectionality Framework, SMW are affected differently by race, ethnicity, socioeconomic status, and geography, leading to varied risk profiles for substance use and disorders. While existing data are largely from high-income nations like the USA, findings suggest that SMW of colour experience greater risks than White SMW, often due to compounded stigma and reduced access to culturally responsive care (Table 2).

**Table 2:** Summary of studies exploring the impact of race, ethnicity, and socioeconomic factors on substance Use risk Among sexual minority women.

Study, Year, Country	Population	Study Sample	Age	SU/SUDs examined	Findings
Mereish & Bradford (2013) (46), USA	Heterosexuals and SMGs	2,556	18–72 years	Lifetime SU problem	SMW of colour had greater risks than White SMW & heterosexual women of colour. No difference in SMM of colour & heterosexual men.
Jeong et al. (2016) (47), USA	Latina, African American, and white SMW	700	≥18 years	Alcohol dependence	Lesbian and bisexual women had high levels of alcohol dependence (Latina, African American > White SMW)
Slater et al. (2017) (35), USA	Gay/lesbian, bisexual, or unsure adults	1,351	≥18 years	Association of discrimination with alcohol use & SUD	High risk of alcohol use among bisexuals & less literate. Increased risk of any SUD among Hispanics
Freitag et al. (2021) (48), USA	Heterosexuals, gay/lesbian, bisexual, and conflicting adults	35,981	≥18 years	Alcohol, tobacco, marijuana use	Bisexual women and racial minorities (Blacks, Hispanics) had highest prevalence of any SUD

Beyond additive disadvantage, emerging scholarship suggests that substance use disparities among SMW may be better conceptualized through a syndemic framework, wherein multiple co-occurring psychosocial and structural adversities interact synergistically rather than independently to worsen health outcomes. In this context, stigma related to sexual orientation may intersect with sexism, racism, poverty, trauma exposure, and mental health burden to create mutually reinforcing pathways toward substance use vulnerability. For example, racial/ethnic minority SMW may simultaneously experience heterosexism within their ethnic communities and racial discrimination within LGBTQ + spaces, compounding stress exposure and reducing access to affirming support networks.

Importantly, these intersecting disadvantages should not be viewed as isolated risk factors but rather as interacting systems of oppression that may amplify one another. For instance, bisexual women from racial minority backgrounds may experience “double marginalization” due to invalidation from both heterosexual and lesbian/gay communities in addition to racial discrimination, potentially increasing emotional distress, maladaptive coping, and treatment disengagement. Such findings support moving beyond single-axis analyses toward integrated intersectional and syndemic models when conceptualizing substance use disparities among SMW.

Although most available evidence originates from the United States, emerging data from LMICs suggest that substance use disparities among sexual and gender minority populations may also extend beyond high-income settings. For instance, Figueiredo et al. (2025), in a Brazilian population-based cohort study of LGBTQIAPN + youth, reported elevated rates of substance use initiation and polysubstance involvement among sexual and gender minority participants compared with heterosexual/cisgender peers, suggesting that these disparities may persist across diverse sociocultural environments despite contextual variation in stigma and healthcare systems (49). However, sex-disaggregated and SMW-specific analyses remain limited in such settings.

Overall, intersectional research highlights that multiple layers of disadvantage, such as being female, a racial/ethnic minority, and a sexual minority, magnify vulnerability to substance use and limit access to protective social resources. Yet, there remains a striking lack of data from low- and middle-income countries, including India, underscoring a major global research gap.

### 3.8 Treatment access and barriers

Compared to their heterosexual counterparts, SMW participate in greater rates of substance use treatment (4), but recent research has raised concerns about how well substance use treatment systems meet the needs of SMW (50).

When seeking addiction treatment, SMW frequently encounter various barriers, the most pertinent of these being interpersonal and institutional stigma. According to Benz et al (51). (2019), stigma is context-dependent in treatment behaviour. They reported that while stigma related to sexual/gender minorities and substance use both affect intentions to seek help, stigma related to substance use was the only factor that predicted actual help-seeking.

Another potent barrier is the geographical and organizational concentration of specific programs tailored to LGBTQ+. According to Cochran et al.'s 2007 (52) survey of SAMHSA (Substance Abuse and Mental Health Services Administration) treatment facilities in the USA, approximately 11.8% of 911 treatment programs claimed to provide specialized services for LGBT clients. But 70.8% of them lacked services tailored to this community. Few (7.4%) provided programs specifically for these clients, and others merely identified as “accepting” without offering specialized care. In another review years later, Williams & Fish (2020) found that only 17.6% of U.S. facilities for substance use treatment offer LGBT-specific programs, with availability higher in private, secular, outpatient/inpatient settings and lower in publicly funded or religious facilities, revealing major gaps in culturally competent care (53).

What also contributed to the barriers that reduced access to treatment were the gaps in provider knowledge and training about the specific issues related to the sexual minorities. Many held negative or ambivalent attitudes towards SMW (especially bisexuals), lowering the perceived safety for disclosure of sexual identity and treatment satisfaction (50).

As per a review by Green & Feinstein (2012) (22), up until 2012, there were very few outcome studies that looked at LGB-specific substance use interventions. These studies were all centred on gay and bisexual men, primarily focused on drug use rather than alcohol use, and did not include SMW. Overall, research showed that common evidence-based interventions like motivational interviewing, contingency management, and cognitive behavioural therapy (CBT) work well for LGB people. There was little evidence that culturally specific protocols for LGB people provide further long-term benefits.

A systematic review in 2021 (54) evaluated all substance use interventions developed or adapted for SMW. Among the four interventions empirically tested included behavioural couples therapy (BCT) (55), mindfulness (56), partner involvement in treatment (57) and contingency management (CM) (58). Out of these four, behavioural intervention (BCT and CM) showed the strongest support. Partner inclusion had mixed results. The only intervention that explicitly incorporated identity-focused (LGBTQ+) adaptation was BCT.

Considering that in programs that were LGBTQ + friendly, it was mostly the intake and assessment process that used inclusive language, but the therapies for this community were not adapted with a preview of the specific challenges they had to face, this review identified four key sexual orientation–specific adaptations for effective substance use treatment for SMW: it should be identity-affirming, grounded in cultural competence and humility, foster community connection and support, and be reinforced by inclusive institutional policies. The only gender-specific adaptation suggested was offering clients the option of a female treatment provider. Minority stress–specific adaptations emphasized addressing minority stress risk factors, strengthening adaptive coping, and integrating trauma-informed approaches to promote safe and empowering care (54).

### 3.9 Research gaps and future directions

Despite growing recognition of substance use disparities among SMW, significant research and implementation gaps remain.

Much of the available literature remains cross-sectional and predominantly U.S.-based, although emerging evidence from LMIC settings such as Brazil (49) suggests similar disparities may exist internationally; nevertheless, SMW-specific data outside Western contexts remain scarce. Further, these studies are solely dependent on self-reported substance use measures, with variable definitions of sexual orientation and substance-related outcomes (54, 59). These features limit causal interpretation and cross-context generalizability. Bisexual, racial/ethnic minority, and rural SMW continue to be significantly underrepresented, and few interventions specifically incorporate minority stress, stigma, or trauma-informed frameworks (50). Furthermore, there are differing definitions of “LGBT-specific” care; many inclusive programs lack significant modifications (52, 53), which makes it challenging to determine the actual scope or significance of LGBT-sensitive care.

Future studies should concentrate on creating and thoroughly evaluating interventions tailored to SMWs, embracing intersectional designs, and creating standardized assessments of LGBT cultural competency. To find out how affirming practices affect engagement, retention, and recovery, longitudinal and implementation studies are required. Barriers to equitable service delivery can be identified at the systems level through structural analyses of funding, policy, and organizational aspects. Enhancing treatment access and outcomes for SMW and the larger LGBT community will require developing a stronger body of evidence supporting stigma-informed, trauma-integrated, and scalable care models.

### 3.10 Limitations of the review

This review has several limitations. First, it is a mini-review rather than a full systematic review or meta-analysis, and therefore may not have captured all eligible studies. The search strategy, while structured, was not prospectively registered and therefore may be subject to selection bias. Second, no formal risk-of-bias instrument was applied. Third, the evidence base is heavily weighted toward U.S. populations, limiting transferability to low- and middle-income settings. Fourth, heterogeneity in sexual orientation definitions, sampling methods, and substance use outcomes complicates direct comparison across studies. Additionally, the absence of prospectively recorded study selection metrics reflects the narrative design of the review and may limit reproducibility. These limitations should be considered when interpreting the conclusions.

## 4 Conclusion

In conclusion, the combined effects of intersectional disadvantage, minority stress, and limited access to culturally competent treatment lead to disproportionate substance use and associated harms among SMW. Theoretical and empirical evidence together highlight the critical need for integrated, identity-affirming, and trauma-informed approaches, even though epidemiological data clearly show higher prevalence rates. Reducing these disparities and enhancing SMW's general well-being will require closing the research-to-practice gap through intersectional frameworks, inclusive policies, and contextually tailored interventions. Future work should prioritize multinational longitudinal studies to clarify culturally specific vs. universal determinants of substance use disparities among SMW.
